# A Phase 2a, Multicenter, Randomized, Double-Blind, Parallel-Group, Placebo-Controlled Trial of IBD98-M Delayed-Release Capsules to Induce Remission in Patients with Active and Mild to Moderate Ulcerative Colitis

**DOI:** 10.3390/cells8060523

**Published:** 2019-05-30

**Authors:** Gionata Fiorino, Giacomo Carlo Sturniolo, Fabrizio Bossa, Andrea Cassinotti, Antonio Di Sabatino, Paolo Giuffrida, Silvio Danese

**Affiliations:** 1 Division of Gastroenterology, IBD Center, Humanitas Clinical and Research Center, 20089 Milan, Italy; gionataf@gmail.com; 2 Department of Biomedical Sciences, Humanitas University, 20090 Milan, Italy; 3 Azienda Ospedaliera di Padova, 35128 Padova, Italy; gc.sturniolo@unipd.it; 4 Fondazione Casa Sollievo della Sofferenza IRCCS—Gastroenterology Department, 71013 Foggia, Italy; f.bossa@icloud.com; 5 UOC Gastroenterologia, ASST Fatebenefratelli Sacco, Luigi Sacco University Hospital, 20157 Milano, Italy; andreacassinotti@libero.it; 6 Dipartimento Area Medica S.C. Medicina Generali, IRCCS Policlinico San Matteo, 27100 Pavia, Italy; A.DiSabatino@smatteo.pv.it (A.D.S.); paolo.giuffrida01@universitadipavia.it (P.G.)

**Keywords:** IBD98-M, sodium hyaluronate, mesalazine, mesalamine, 5-ASA, ulcerative colitis, inflammatory bowel disease

## Abstract

IBD98-M is a delayed-release formulation of mesalamine (mesalazine) and SH with a potential therapeutic role in ulcerative colitis (UC). A total of 51 patients with a modified Ulcerative Colitis Disease Activity Index (UCDAI) score of ≥4 and ≤10, and a modified UCDAI endoscopy subscore ≥1 were randomized for 6 weeks of double-blind treatment with IBD98 0.8 g/day or IBD 1.2 g/day or placebo. The efficacy and safety of IBD98-M in mild to moderate active UC were primarily evaluated. At week 6, 1 (5.9%), 2 (12.5%), and 2 (11.1%) patients receiving IBD98-M 0.8 g, IBD98-M 1.2 g, and placebo, respectively, (*p* > 0.999) achieved clinical remission. Higher clinical response was seen in IBD98-M 1.2 g (31.3%) versus placebo (16.7%) and endoscopic improvement in IBD98-M 0.8 g (29.4%) versus placebo (22.2%) was seen. Fecal calprotectin levels were reduced in IBD98-M groups versus placebo (*p* > 0.05). IBD98-M patients achieved significant improvement in physical health summary score component of the SF-36 (*p* = 0.01 and *p* = 0.03 respectively) compared to placebo. IBD98-M did not meet the primary end point but had higher clinical response (1.2 g/day) and endoscopic improvement (0.8 g/day) compared to placebo. The safety result shown that IBD98-M treatment was safe and well tolerated in this patient population. No new safety signals or unexpected safety findings were observed during the study. Further trials with different stratification and longer follow-up may be needed to evaluate the efficacy.

## 1. Introduction

Ulcerative colitis (UC) is a chronic, relapsing inflammatory bowel disease (IBD) affecting the colon. The disease usually involves the rectum but may extend proximally to involve a portion of or the entire colon [1]. About 40% to 50% of patients have disease that is limited to the rectum and the recto-sigmoid colon, 30% to 40% have disease extending beyond the sigmoid flexure but not involving the whole colon, and 20% have pancolitis [2]. The mainstay of therapy for mild to moderate UC is sulfasalazine and 5-aminosalicylic (5-ASA) agents. These agents are effective at inducing remission in UC and in maintaining remission in UC [1,3]. However, when 5-ASA compounds fail in inducing and/or maintaining disease remission, UC patients need to escalate to steroid-based therapies which is effective but limited by serious adverse events in almost half of patients [4]. Two meta-analyses of induction studies concluded that the mean remission rate with mesalamine was 42%, compared with 24% in placebo-treated patients [5,6]. Other two meta-analyses determined that from 58.7% to 72% of UC patients do not achieve remission [7,8]. Therefore, giving an alternative to steroids in mild-to-moderate disease with significantly better safety profile would be of additional value for these patients.

Sodium hyaluronate, the sodium salt of hyaluronic acid (HA), functions as a tissue lubricant and is thought to play an important role in modulating the interactions between adjacent tissues [9]. Sodium hyaluronate may enhance the mucosal repair and restoration of the mucosal protective layer. In facilitating wound healing [10], it is thought that sodium hyaluronate acts as a protective transport vehicle, taking peptide growth factors and other structural proteins to a site of action. HA has shown clinical effectiveness in several conditions where wound repair was the main therapeutic goal [10,11,12,13,14,15,16,17]. Based on these properties, it is expected that supplementation of sodium hyaluronate may provide a protective barrier to the lining of the colon affected by UC [18].

Combining 5-ASA and HA in a unique oral formulation might increase the probability to induce clinical remission compared to placebo and add one more steroid-free alternative to mild-to-moderate UC patients. The combination of mesalamine with sodium hyaluronate may also allow a reduction in dose of mesalamine, to minimize side effects and enhance therapeutic efficacy. Based on this hypothesis, the primary aim of this trial was to test the efficacy and safety profile of a combined formulation of the two active principles in this patients’ setting.

## 2. Methods

A Phase 2a, multicenter, randomized, double-blind, parallel-group, placebo-controlled trial in patients with active, mild to moderate UC (IBD98-M-2002) [3]. It was conceived as an exploratory proof-of-concept study to investigate the clinical efficacy of IBD98-M delayed-release capsules (in a fixed combination) over a 6-week treatment period and a 2-week follow-up period.

### 2.1. Study Drug

IBD98-M is a delayed-release formulation, in the form of a capsule filled with enteric coated pellets designed to allow the release of both mesalamine and sodium hyaluronate at pH 6.8 or above at the terminal ileum (ascending colon). Each capsule is filled with multiple-layer pellets containing 200 mg of mesalamine and 23 mg of sodium hyaluronate. Each pellet consists of a microcrystalline cellulose inner core, which is coated first with mesalamine, then with sodium hyaluronate, and finished with an enteric coating film. The safety and efficacy of sodium hyaluronate enema (IBD98E, a solution formulation of sodium hyaluronate for local application) for the treatment of mild to moderate UC was investigated in humans following self-administration at bed time for 28 days, with promising results in terms of clinical response and remission, and mucosal healing [19].

### 2.2. Study Objectives

The primary objective of this study was to compare the percentage of patients in UC remission, defined as the modified Ulcerative Colitis Disease Activity Index (UCDAI) score of ≤1, with a score of 0 for rectal bleeding and stool frequency, no mucosal friability, and sigmoidoscopy score not exceeding 1, at Week 6 for each of the two IBD98-M dose groups compared with those patients receiving placebo.

Secondary Objectives were to compare clinical response rates at Week 6 among treatment groups, defined as a ≥3 point reduction from baseline in the modified UCDAI score, to compare endoscopic improvement at Week 6 among treatment groups (defined as a ≥1 point decrease in modified UCDAI mucosal appearance subscore), to determine and compare the change in symptoms (rectal bleeding and stool frequency) from baseline to each study visit within treatment groups, and to evaluate the safety and tolerability profile of IBD98-M. Exploratory outcomes were to investigate the effect of IBD98-M treatment on the levels of fecal calprotectin (FC), and to examine any correlation between therapeutic response to IBD98-M and baseline characteristics of the study patients.

### 2.3. Study Design and Procedures

Patients were screened for study enrollment up to 2 weeks prior to randomization. During the screening period, patients were evaluated with laboratory tests to confirm active inflammation and exclude active concomitant intestinal infections, physical examinations, and sigmoidoscopy. To be eligible, patients must have a modified UCDAI score of ≥4 and ≤10 and a score of ≥1 on the endoscopy subscore. In addition, the diagnosis of UC had to be confirmed visually by endoscopy images and also by histologic evidence in the past. Other inclusion criteria were age between ≥18 and <75 years, confirmed diagnosis since at least 6 months prior to screening, not with childbearing potential or adopting effective contraception, and able to understand and sign the informed consent. Patients with ulcerative proctitis only (≤15 cm of disease from the anal verge), patients who were pregnant or breastfeeding, patients with active intestinal and/or colonic infection, or needed higher doses of 5-ASA (>2.4 g/day), or with known intolerance or allergy to the study active principles and excipients, or with any health condition at risk of safety during the study period according to the investigator’s judgement were excluded from the study.

After the screening visits, eligible patients were randomized 1:1:1 to receive:

1. IBD98-M 0.8 g/day (mesalamine 0.8 g with sodium hyaluronate 92 mg), or
2. IBD98-M 1.2 g/day (mesalamine 1.2 g with sodium hyaluronate 138 mg), or
3. Placebo.

Study blind was maintained using a double-dummy technique. Eligible patients were randomized centrally via an Interactive Web Response System (IWRS). The randomization code was computer-generated by InVentive Health Clinical (Raleigh, NC, USA) but it was not available to the Bioanalytical Division of inVentive until the clinical and analytical phases of the study were completed.

Each patient received 3 capsules twice a day for a period of 6 weeks, administered orally in one of the following regimens:

- Patients in group 1 received 2 capsules of IBD98-M (200 mg of mesalamine/23 mg of sodium hyaluronate) and 1 placebo BID, a total of 800 mg of mesalamine and 92 mg of sodium hyaluronate.
- Patients in group 2 received 3 capsules of IBD98-M (200 mg of mesalamine/23 mg of sodium hyaluronate) BID, a total of 1200 mg of mesalamine and 138 mg of sodium hyaluronate.
- Patients in the placebo group received 3 placebo capsules BID.

Patients were encouraged to take their medication at the same time, 30–120 min before meals every day.

The study drug was furnished by Holy Stone Healthcare Co. Ltd, (Taipei, Taiwan).

Patients were evaluated during screening and every 2 weeks after randomization up to week 6; then an additional follow-up visits was planned at week 8 as visit 7. Clinical parameters, changes in the medical history and concomitant medications, and relevant laboratory tests were assessed and recorded at each visit according to the study plan. A flexible sigmoidoscopy was required at week 6 or at the early termination visit.

Symptoms and adverse events (AEs) were self-reported by each patient in a daily diary starting at Visit 1 and continuing until the end of treatment (Visit 6/early termination). At each visit, or whenever needed, the symptoms and adverse events were recorded and managed by the investigating physician.

Quality of life was assessed by using the IBD-Questionnaire (IBD-Q) [20] and the Short Form 36 (SF-36) [21].

### 2.4. Ethical Considerations

The study was designed according to the Declaration of Helsinki. The protocol was reviewed and approved by coordinator Ethical Committee (Comitato Etico Indipendente Humanitas Clinical and Research Center, authorization number 1478) then agreed by each local Ethical Committee of 12 participating centers. The approved informed consent forms were obtained from participated patients prior to randomization.

### 2.5. Statistical Methods

#### Sample Size Calculation

Simon’s randomized Phase 2 design was used [22,23,24]. The average remission rate of placebo in this study population was estimated at approximately 20% [25]. Assuming a power of 80%, and difference in remission rates between the best treatment and the other treatments ≥18%, we calculated that each arm required 17 patients, giving the total number of patients to be enrolled as 51. This enrollment was estimated to provide 28.2% precision with a 95% confidence interval (CI) for evaluation of the difference between study treatment and placebo, where precision was defined as ½ width of the confidence interval.

The statistical evaluation was performed using Statistical Analysis Software (SAS®) Version 9.4 or higher (SAS Institute, Cary, NC, USA).

### 2.6. Analysis Populations

For continuous variables, data were summarized with the number of patients, mean, standard deviation (SD), median, minimum, and maximum by treatment group. For categorical variables, data were tabulated with the number and percentage of patients for each category by treatment group. The intent-to-treat (ITT) population included all randomized patients who received at least one dose of study medication irrespective of any deviation from the protocol or premature discontinuation. The treatment group assignment was designated according to initial randomization. The ITT population served as the basis for the analysis of efficacy. A per-protocol population (PP) analysis was also performed on all patients who did not violate the terms of the protocol in a way that would impact the study outcome significantly, as determined by the Medical Monitor. All decisions to exclude patients from the per-protocol population dataset was made prior to the unblinding of the study [26].

The safety population included all randomized patients who receive at least 1 dose of study drug. The treatment group assignment in this population was defined by the treatment actually received.

Treatment groups were compared using Fisher’s exact test [27]. Each test was 2-sided and declared statistically significant at the 0.05 level. As a supportive analysis, the primary analysis was also done using the per protocol population. Also, sensitivity analysis to assess the effects of missing data was performed [28]. Generalized estimating equations (GEEs) were used to assessed the association of interests, as well to adjust the correlation arising from repeated measurements [29]. Differences in the outcome measures between baseline and week 6 were assessed using ANCOVA test [30].

All reported AEs were coded using the Medical Dictionary for Regulatory Activities, (MedDRA, McLean, VA, USA) Version 21.0. The incidence of treatment emergent adverse events (TEAEs) was summarized. Events with missing onset dates were managed as treatment-emergent. If a patient experienced more than 1 occurrence of the same AE, the occurrence with the greatest severity and the closest association with the study drug were used in the summary tables. SAEs and AEs causing discontinuation will be tabulated. All AEs were collected by patient, along with information regarding onset, duration, severity, and relationship to study drug, action taken with study drug, treatment of event, and outcome. Clinical laboratory data and vital signs were summarized using descriptive statistics including mean values and mean change from baseline values, as well as numbers of patients with values outside limits of the normal range at each time point. No interim analysis was planned.

## 3. Results

**Figure 1:**
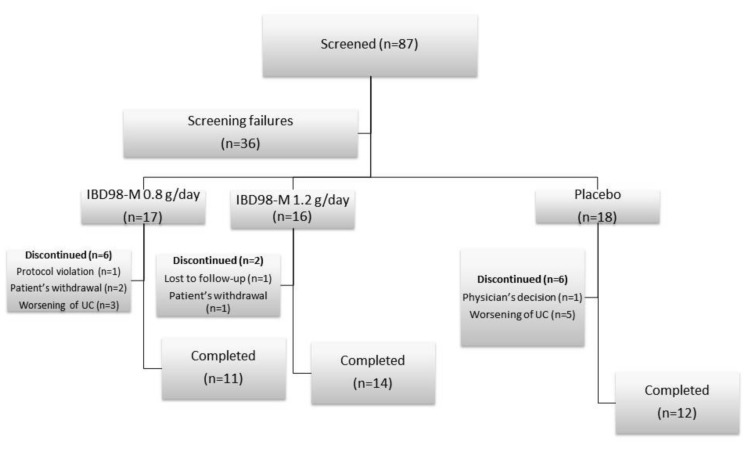
Distribution of the study population.

From 28 Jan 2016 to 02 Jul 2018, 87 patients in 13 centers were screened, and 51 patients were finally randomized (Figure 1).

Of these patients, 37 (72.5%) completed the study. Baseline characteristics of patients are summarized in Table 1.

At week 6, clinical remission (primary endpoint) was achieved in 1 (5.9%), 2 (12.5%), and 2 (11.1%) patients in the IBD98-M 0.8 g/day group, the IBD98-M 1.2 g/day group, and the placebo group, respectively (Table 2). No statistically significant difference was observed between the treatment groups (*p* > 0.999), both at the ITT and PPP analysis. No statistically significant differences were observed when clinical responses in the active treatment groups were compared to placebo. Clinical response was achieved by 3 (17.6%), 5 (31.3%), 3 (16.7%) patients in the IBD98-M 0.8 g/day group, the IBD98-M 1.2 g/day group, and the placebo group, respectively. Differences between the treatment groups and the placebo group were not statistically significant (Table 3). A numerically higher proportion of patients with endoscopic improvement was found in the IBD98-M 0.8 g/day group (*n* = 5, 29.4%) compared with the IBD98-M 1.2 g/day group (*n* = 2, 12.5%) and the placebo group (*n* = 4, 22.2%), however no statistically significant difference was observed between groups; IBD98-M 0.8 g/day group and the placebo group (*p* = 0.683) or the IBD98-M 1.2 g/day group and the placebo group (*p* = 0.648) (Table 4).

The normal distribution test for UCDAI as well as endoscopic scores were done using SAS Proc univariate (Cary, NC, USA), and the data resulted not having normal distribution.

Differences in the total UCDAI and endoscopic scores between baseline and week 6 using ANCOVA test are shown in Table 5 and Table 6.

Least square means difference in fecal calprotectin levels between the IBD98-M 0.8 g/day group and the placebo group was 31.4 (332.87) and the IBD98-M 1.2 g/day group and the placebo group was 160.3 (386.92). This difference was not statistically significant for the IBD98-M 0.8 g/day (*p* = 0.926) group or the IBD98-M 1.2 g/day group (*p* = 0.683) and the placebo group.

Quality of life (measured by the IBDQ and SF-36) resulted improved in patients receiving any dose of study drug compared to placebo. At visit 6, median IBDQ score increased from baseline for the IBD98-M 0.8 g/day group (5.0) and the IBD98-M 1.2 g/day group (13.0) and decreased for the placebo group (2.0). Difference between the IBD98-M 0.8 g/day group (*p* = 0.257) and the IBD98-M 1.2 g/day group (*p* = 0.182), versus placebo group was not statistically significant. For physical health summary score component of the SF-36, statistically significant difference was observed between the IBD98-M 0.8 g/day group and the placebo group (*p* = 0.010) and between the IBD98-M 1.2 g/day group and the placebo group (*p* = 0.032). For other 2 components, difference between the placebo group and the treatment groups was not statistically significant.

### Safety

During the entire study period, AEs were reported for 10 patients (58.8%) in the IBD98-M 0.8 g/day group, 11 patients (68.8%) in the IBD98-M 1.2 g/day, and 15 patients (83.3%) in the placebo group. Overall, 7 patients (13.7%) reported drug-related AEs. Severe AE was reported in 1 patient (5.6%) in the placebo group.

Overall safety population of TEAE by treatment group were reported for 33 patients (64.7%), of that 10 patients (58.8%) were in the IBD98 M 0.8 g/day group, 10 patients (62.5%) were in the IBD98-M 1.2 g/day, and 13 patients (72.2%) were in the placebo group. TEAEs leading to discontinuation of study drug were reported for 6 patients (11.8%). The study drug was discontinued due to related TEAE in 1 patient (2.0%) in the IBD98-M 0.8 g/day group.

TEAEs reported in ≥2 patients are summarized and categorized by System Organ Class (SOC) and Preferred Terms (PT) below. Overall, 33 patients (64.7%) reported at least 1 TEAE. The proportion of patients reporting at least 1 TEAE was higher in the placebo group (72.2%), compared with the IBD98-M 1.2 g/day group (62.5%), and the IBD98-M 0.8 g/day (58.8%) group. Most commonly reported TEAE was headache, reported in 11 patients (21.6%), followed by ulcerative colitis reported in 8 patients (15.7%).

Most commonly reported drug-related TEAE by worst relationship, SOC and PT included nausea and headache, reported in 2 patients (3.9%), each. Other related TEAEs included dyspepsia, diarrhea, pyrexia, and dizziness, reported in 1 patient (2.0%), each.

Only 1 SAE was reported in a screen failure subject. No SAE was reported during the study. No serious TEAEs were reported during the study.

The study drug was discontinued due to TEAE for 4 patients (23.5%) in the IBD98-M 0.8 g/day group and for 2 patients (11.1%) in the placebo group. No discontinuation for adverse event was reported for any patient in the IBD98-M 1.2 g/day group. Overall, the most commonly reported TEAE that led to study drug discontinuation or withdrawal from study was ulcerative colitis reported for 4 patients (7.8%). No AE leading to early withdrawal from the study was reported. No death was reported during the study.

## 4. Discussion

HA has been successfully used in medical conditions where wound repair is required, such as interstitial cystitis, perioral wrinkles, joint lesions, and nasal wounds [10,11,12,13,15,16,17]. In all these studies, patients treated with local administration of HA achieved wound repair and tissue restoration. One study investigated the efficacy and safety of HA enemas in 21 patients with mild-to-moderate UC limited to the distal tract, showing a clinical remission rate of 38% and an endoscopic remission rate of 47.6% at week 4 [19].

This is the first study investigating the efficacy and safety profile of HA administered orally as an add-on strategy to mesalamine. In our patients’ population, the administration of IBD98-M 0.8 g/day and 1.2 g/day did not result in statistically significantly higher proportions of patients achieving clinical and endoscopic response and remission. We observed only significant benefits in terms of the physical health summary scores as assessed by the SF-36. However, we found encouraging signals in favor of HA treatment in our study population. The rates of clinical and endoscopic improvement measured by the UCDAI were numerically higher in both treatment groups and showed a possible dose-dependent beneficial effect. Moreover, the group treated with IBD98-M 1.2 g/day showed the lowest rate of early withdrawal during the study period (12.5% vs. 33% treated with placebo).

Due to the differences of profile of the patients per group, further investigation using GEE (generalized estimating equations) statistics method showed that there were significant changes in the UCDAI scores from the first to last visit within each treatment arm (0.8 g/day *p* = 0.0283, 1.2 g/day = 0.0132, Placebo *p* = 0.2939). GEE statistics also showed that within in the 1.2 g/day group, there were statistically significant changes at each visit for the patients rectal bleeding scores from baseline to final visit (*p* = 0.0037). This was not seen in 0.8 g/day *p* = 0.4980 and placebo *p* = 0.9139). There were also statistically significant changes within the 1.2 g/day group in stool frequency from baseline to final visit (*p* = 0.0070). This was not seen in 0.8 g/day (*p* = 0.3262) and placebo (*p* = 0.8038).

No treatment-related serious adverse events were reported during the study, and no differences in AEs were observed between the study drug and placebo, supporting the very good safety profile of IBD98-M.

There are some possible reasons to explain our data and the analyses done with GEE statistics, and some limitations of the present study. First, the requirement to withdraw any background stable therapy with 5-ASA at least for 2 weeks before the baseline visit was maybe too extreme. This may have led to a possible worsening in disease activity that could not be effectively treated by the lower dose of IBD98-M (0.8 g/day). This explains why there was less reduction in the UCDAI score in this subgroup and more early withdrawals. Second, both 1.2 g and 0.8 g study groups showed a time-dependent decrease in the UCDAI score up to week 6, whereas patients with placebo showed no further improvement after week 3. This may suggest that administering IBD98-M for longer than 6 weeks may result in significantly differences in terms of response and remission compared to placebo. This is observed as well in the improved group using median UCDAI change (without endoscopic scores) with 1.2 g/day group having the largest decrease in score (−4.2) vs. 0.8 g/day and Placebo (−1.17 and −1.8 respectively). This assumption needs further investigations in further clinical trials. Third, the general distribution of the data was not normal, probably due to the relatively small sample size, and the distribution of patients with UCDAI 9–10 was higher in the IBD98-M treated group whereas no patients with this grade of severity were allocated in the placebo group. Despite this unbalanced distribution in disease severity, we observed up to 40% of patients scored 9–10 who achieved improvement from the 1.2 g daily dose.

## 5. Conclusions

This was the first attempt to evaluate efficacy and safety of oral formulation of sodium hyaluronate in UC patients with mild-to-moderate active disease. Despite our data did not show statistically significant superiority of IBD98-M towards placebo in terms of primary endpoints of efficacy (clinical response and remission, endoscopic response and remission), IBD98-M showed significant reduction in biomarkers of inflammation (fecal calprotectin) and significant improvement of quality of life. The potential role of IBD98-M in treating mild-to-moderate UC is worthy of further investigation. Further clinical trials with longer time of exposure to IBD98-M are needed.

## Figures and Tables

**Table 1:** Baseline characteristics of patients.

	IBD98-M 0.8 g/day (n = 17) n (%)	IBD98-M 1.2 g/day (N = 16) n (%)	Placebo (N = 18) n (%)	Total (N = 51) n (%)
Age (years), Mean ± SD	36.6 ± 10.68	43.1 ± 12.23	45.8 ± 12.21	41.9 ± 12.14
Males	12 (70.6)	7 (43.8)	9 (50.0)	28 (54.9)
Current smokers	0	1 (6.3)	0	1 (2.0)
BMI at Screening (kg/m^2^), Mean ± SD	24.08 ± 3.104	25.22 ± 3.771	24.38 ± 4.016	24.54 ± 3.614
Baseline UCDAI				
≤5	7 (43.8)	7 (43.8)	9 (50.0)	23 (45.1%)
6–8	5 (31.3)	4 (25.0)	9 (50.0)	18 (35.2%)
9–10	4 (25.0)	5 (31.3)	0 (0.0)	9 (17.7%)
Mean endoscopic score	2.08	1.79	1.86	1.90
Concomitant medications				
Agents acting on the renin-angiotensin system	1 (5.9)	0	3 (16.7)	4 (7.8)
Ace inhibitors	1 (5.9)	0	2 (11.1)	3 (5.9)
Ramipril	0	0	2 (11.1)	2 (3.9)
Analgesics	5 (29.4)	5 (31.3)	5 (27.8)	15 (29.4)
Anilides	5 (29.4	5 (31.3)	5 (27.8)	15 (29.4)
Paracetamol	5 (29.4)	5 (31.3)	5 (27.8)	15 (29.4)
Antianemic preparations	1 (5.9)	1 (6.3)	0	2 (3.9)
Iron, parenteral preparations	1 (5.9)	1 (6.3)	0	2 (3.9)
Ferric carboxymaltose	1 (5.9)	1 (6.3)	0	2 (3.9)
Antibacterials for systemic use	2 (11.8)	1 (6.3)	2 (11.1)	5 (9.8)
Penicillins, incl. Beta-lactamase inhibitors	1 (5.9)	0	1 (5.6)	2 (3.9)
Amoxicillin + clavulanic acid	1 (5.9)	0	1 (5.6)	2 (3.9)
Antidiarrheals, intestinal anti-inflammatory/anti-infectious agents	6 (35.3)	7 (43.8)	7 (38.9)	20 (39.2)
Aminosalicylic acid and similar agents	4 (23.5)	7 (43.8)	7 (38.9)	18 (35.3)
Mesalazine	4 (23.5)	7 (43.8)	7 (38.9)	18 (35.3)
Antidiarrheal microorganisms	2 (11.8)	0	2 (11.1)	4 (7.8)
Corticosteroids acting locally	2 (11.8)	1 (6.3)	1 (5.6)	4 (7.8)
Prednisone	2 (11.8)	0	1 (5.6)	3 (5.9)
Anti-inflammatory and anti-rheumatic products	2 (11.8)	2 (12.5)	2 (11.1)	6 (11.8)
Propionic acid derivatives	2 (11.8)	1 (6.3)	2 (11.1)	5 (9.8)
Ketoprofen	2 (11.8)	1 (6.3)	0	3 (5.9)
Ibuprofen	0	0	2 (11.1)	2 (3.9)
Cough and cold preparations	1 (5.9)	0	2 (11.1)	3 (5.9)
Other cough suppressants	1 (5.9)	0	1 (5.6)	2 (3.9)
Drugs for acid related disorders	1 (5.9)	1 (6.3)	2 (11.1)	4 (7.8)
Proton pump inhibitors	1 (5.9)	1 (6.3)	1 (5.6)	3 (5.9)
Lansoprazole	1 (5.9)	0	1 (5.6)	2 (3.9)
Drugs for constipation	4 (23.5)	6 (37.5)	3 (16.7)	13 (25.5)
Enemas	4 (23.5)	6 (37.5)	2 (11.1)	12 (23.5)
Fleet	1 (5.9)	4 (25.0)	1 (5.6)	6 (11.8)
Sodium phosphate	2 (11.8)	1 (0.3)	1 (5.6)	4 (7.8)
Mineral supplements	1 (5.9)	1 (6.3)	1 (5.6)	3 (5.9)
Calcium, combinations with vitamin d and/or other drugs	1 (5.9)	1 (6.3)	1 (5.6)	3 (5.9)
Calcium w/colecalciferol	1 (5.9)	0	1 (5.6)	2 (3.9)
Psycholeptics	2 (11.8)	1 (6.3)	1 (5.6)	4 (7.8)
Benzodiazepine derivatives	1 (5.9)	0	1 (5.6)	2 (3.9)
Benzodiazepine derivatives	1 (5.9)	1 (6.3)	0	2 (3.9)

Abbreviation: IBD: inflammatory bowel disease; SD = standard deviation; UCDAI = Ulcerative Colitis Disease Activity Index. Percentages were calculated based on the number of patients in the safety population in each treatment group.

**Table 2:** Primary efficacy analysis: percentage of patients in remission at week 6 (ITT population).

		IBD98-M 0.8 g/day (N = 17)	IBD98-M 1.2 g/day (N = 16)	Placebo (N = 18)
**Remission at Week 6**	n (%)	1 (5.9)	2 (12.5)	2 (11.1)
	95% confidence interval	0.1, 28.7	1.6, 38.3	1.4, 34.7
	*p* value versus placebo	>0.999	>0.999	

Abbreviations: ITT = intent-to-treat; UCDAI = Ulcerative Colitis Disease Activity Index.

**Table 3:** Proportion of patients with clinical improvement at week 6 (ITT Population).

		IBD98-M 0.8 g/day (N = 17)	IBD98-M 1.2 g/day (N = 16)	Placebo (N = 18)
**Clinical Improvement at Week 6**	n (%)	3 (17.6)	5 (31.3)	3 (16.7)
	95% confidence interval	5.5, 57.2	12.8, 64.9	4.7, 50.8
	*p* value versus placebo	>0.999	0.678	

Abbreviations: ITT = intent-to-treat; UCDAI = Ulcerative Colitis Disease Activity Index.

**Table 4:** Proportion of patients with endoscopic improvement at week 6 (ITT Population).

		IBD98-M 0.8 g/day (N = 17)	IBD98-M 1.2 g/day (N = 16)	Placebo (N = 18)
**Endoscopic improvement at Week 6**	n (%)	5 (29.4)	2 (12.5)	4 (22.2)
	95% confidence interval	15.2, 72.3	1.8, 42.8	8.4, 58.1
	*p* value versus Placebo	0.683	0.648	

Abbreviations: ITT = intent-to-treat; UCDAI = Ulcerative Colitis Disease Activity Index.

**Table 5:** Total UCDAI score (patient including withdrawn with completed UCDAI score).

	IBD98-M 0.8 g/day (N = 12)	IBD98-M 1.2 g/day (N = 14)	Placebo (N = 14)
UCDAI at Baseline			
n	12	14	14
Median (range)	5.00 (4–10)	5.50 (4–10)	5.00 (4–8)
UCDAI at Visit 6			
n	12	14	14
Median	4.00 (1–9)	4.50 (0–11)	5.00 (0–10)
UCDAI at Visit 6 – Baseline			
n	12	14	14
Median	−1.00 (−4–3)	−1.00 (−5–1)	−1.00 (−4–6)
*p* value *	0.837	0.335	

*p value comparing treatment groups (IBD98 vs. placebo) using ANCOVA. Since the population was small, and the distribution was not normal, the median was used to show the changes in the endoscopic subscore.

**Table 6:** Changes of endoscopic subscore (Patient including withdrawn with completed UCDAI score).

	IBD98-M 0.8 g/day (N = 12)	IBD98-M 1.2 g/day (N = 14)	Placebo (N = 14)
Endoscopic score at baseline			
n	12	14	14
Median (range)	2.00 (1–3)	2.00 (1–3)	2.00 (1–3)
Endoscopic score at visit 6			
n	12	14	14
Median (range)	1.50 (0-3)	2.00 (0–3)	2.00 (0–3)
Endoscopic score at visit 6 – baseline			T
n	12	14	14
Median	0.00 (−2–0)	0.00 (−1–1)	0.00 (−2–1)
*p* value *	0.133	0.832	

*p value comparing treatment groups (IBD98 vs. Placebo) using ANCOVA. Since the population was small, and the distribution was not normal, the median was used to show the changes in the endoscopic subscore.
